# Enhancing Clinical Data Management Through Barcode Integration and Research Electronic Data Capture: Scalable and Adaptable Implementation Study

**DOI:** 10.2196/70016

**Published:** 2025-09-12

**Authors:** Rendong Zhang, Sophie Chiron, Regina Tyree, Kate Carson, Larry Raber, Karthik Ramadass, Chenyu Gao, Michael E Kim, Lianrui Zuo, Yike Li, Zhiyu Wan, Paul A Harris, Qi Liu, Ken S Lau, Lori A Coburn, Keith T Wilson, Yuankai Huo, Bennett A Landman, Shunxing Bao

**Affiliations:** 1 Department of Electrical and Computer Engineering, School of Engineering, Vanderbilt University, PMB 351824, 2301 Vanderbilt Place, Nashville, TN, 37235-1824, United States, 1 6153222338; 2 Vanderbilt-Ingram Cancer Center, Vanderbilt University Medical Center, Nashville, TN, United States; 3 Department of Medicine, Division of Gastroenterology, Hepatology, and Nutrition, Vanderbilt University Medical Center, Nashville, TN, United States; 4 Department of Computer Science, School of Engineering, Vanderbilt University, Nashville, TN, United States; 5 Department of Otolaryngology-Head and Neck Surgery, Vanderbilt University Medical Center, Nashville, TN, United States; 6 School of Biomedical Engineering, ShanghaiTech University, Shanghai, China; 7 Department of Biomedical Informatics, Vanderbilt University Medical Center, Nashville, TN, United States; 8 Department of Biomedical Engineering, Vanderbilt University, Nashville, TN, United States; 9 Department of Biostatistics, Vanderbilt University Medical Center, Nashville, TN, United States; 10 Center for Quantitative Sciences, Vanderbilt University Medical Center, Nashville, TN, United States; 11 Epithelial Biology Center, Vanderbilt University Medical Center, Nashville, TN, United States; 12 Department of Cell and Developmental Biology, School of Medicine, Vanderbilt University, Nashville, TN, United States; 13 Vanderbilt Center for Mucosal Inflammation and Cancer, Nashville, TN, United States; 14 Program of Cancer Biology, School of Medicine, Vanderbilt University, Nashville, TN, United States; 15 VA Tennessee Valley Healthcare System, Nashville, TN, United States; 16 Department of Pathology, Microbiology, and Immunology,, Vanderbilt University Medical Center, Nashville, TN, United States

**Keywords:** clinical data management, barcode technology, REDCap, data granularity, Docker containerization, system throughput optimization

## Abstract

**Background:**

Effective data management is crucial in clinical studies for precise tracking, secure storage, and reliable analysis of samples. Traditional systems often encounter challenges like barcode recognition errors, inadequate data details, and diminished performance under heavy workloads.

**Objective:**

This paper aims to enhance clinical data management by improving barcode robustness, increasing data granularity, and boosting system throughput. These improvements address key challenges in barcode informatics systems, as highlighted in previous studies, to better support real clinical applications. In addition, we aim to validate the design criteria on various gastrointestinal-related studies, ensuring it can be easily integrated into other clinical data management workflows.

**Methods:**

We evaluated the robustness of various barcode technologies under significant blurring conditions, implemented a dynamic organ-specific archive in the REDCap (Research Electronic Data Capture) database for various clinical study data collection criteria, and used Docker to containerize the informatics software for different studies. In addition, we proposed a local cache system to reduce interaction times with REDCap for large-scale data records. Experimental setups include assessing barcode recognition accuracy under various levels of image blurring, showcasing different study types managed with the organ-specific archive, and measuring system throughput and response times with and without the proposed local cache system.

**Results:**

Our findings demonstrate that the DataMatrix barcode exhibits superior resilience, maintaining high recognition accuracy under blurred conditions. The dynamic organ-specific archive in REDCap enabled precise tracking of sample origins, improving data granularity. Docker containerization streamlines software deployment and ensures consistency across studies. The local cache system significantly reduces interaction times with REDCap, decreasing operating time by nearly eightfold compared to the naïve strategy when handling large patient datasets.

**Conclusions:**

The proposed enhancements significantly improve barcode robustness, data granularity, and system throughput in the informatics system, addressing key limitations identified in previous studies. These optimizations ensure efficient data management and robust support for diverse clinical research needs.

## Introduction

In the digital age, operations across various fields are transforming, and digital pathology is revolutionizing traditional histopathological diagnosis by replacing microscopes and glass slides with virtual microscopy on computers [1,2]. Digital pathology has evolved into an exceptionally powerful and precise tool for the diagnosis and prognosis of a wide range of diseases [3-5]. However, hardware and software issues can greatly affect costs and slow down the implementation of clinical data management workflows [6]. Efficiently collecting and using data can be a significant challenge in clinical research due to the complexity and diversity of data types, the need for precise tracking and storage, and the potential for errors and inefficiencies in manual data handling. Therefore, an advanced informatics system for data management is essential and in high demand for ensuring precise and efficient usage of clinical research data [1,7-9]. Integrating tools like barcoding, specimen tracking, and digital dictation enhances the safety, quality, and efficiency of clinical research workflows [10]. Despite the clear potential improvements with a fully digital approach, only a small number of clinical research laboratories have transitioned to such workflows [11].

Clinical studies usually have complex and diverse data management needs that require tailored solutions depending on the nature and objectives of each study. For instance, some studies may need to collect samples from different organs or regions of the body to analyze specific diseases or conditions. Nourani et al [12] assessed various clinical data management systems by analyzing databases such as Web of Science, Scopus, Science Direct, ProQuest, Ovid Medline, and PubMed. Their study concluded that none of the systems comprehensively addressed all data management aspects in clinical trials. Therefore, it is often necessary to create customized data management systems tailored to specific needs. Without robust data management systems, researchers face challenges like data loss, sample tracking errors, and inefficiencies in data retrieval and analysis that compromise study quality and reliability and pose health risks or legal challenges for clinicians [9].

One widely used tool for managing clinical research data is REDCap (Research Electronic Data Capture), a web-based application developed by Vanderbilt University Medical Center [13,14]. REDCap offers a user-friendly interface for validated data entry, audit trails for tracking, and automated data exports for statistical analysis, making it a popular choice among academic, nonprofit, and government institutions worldwide [15-19]. Building on the capabilities of REDCap, Bao et al [20] designed a cross-platform informatics system with two key features: (1) a digitalized and human-readable barcode for sample tracking using a cost-effective printer, and (2) longitudinal archive storage in REDCap. After identifying several real-life bottlenecks, which are introduced below, we extended this system to more clinical studies over the past 5 years, including Crohn disease Gut Cell Atlas, Translational Impact of Electrophile Adducts in Colorectal Cancer. The project includes normal control, ulcerative colitis, Crohn disease, and colorectal cancer. It also includes surgical resections from the Cooperative Human Tissue Network in addition to colonoscopy biopsies. *Helicobacter pylori* (*H. pylori*)-associated gastric inflammation and disease progression, and gut–microbiome-related gastrointestinal and liver diseases, demonstrating significant improvements in data handling efficiency.

**Figure 1.**
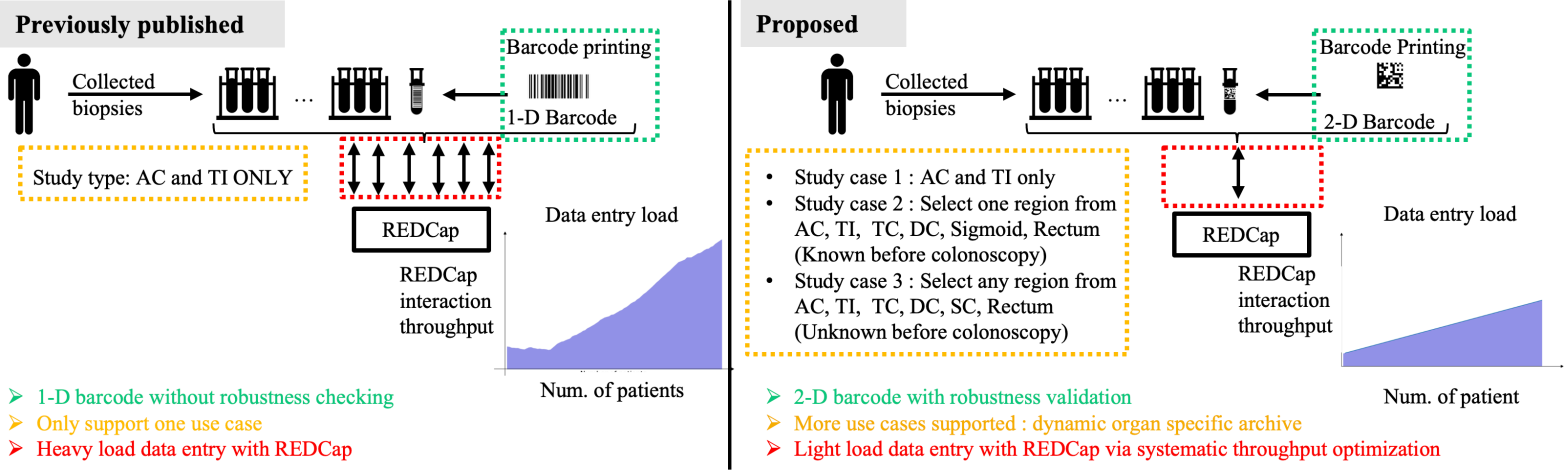
Comparison of the published and proposed barcode systems for collected biopsies. The published system uses 1-D barcodes, supports only one use case (ascending colon [AC] and terminal ileum [TI]), and involves a heavy data entry load with REDCap without robustness checking. In contrast, the proposed system uses 2-D barcodes with robustness validation, supports multiple use cases, including dynamic organ-specific archives, and optimizes data entry with a lighter load on REDCap. For example, Study 1 covered that the published system collects samples only from the AC and TI. In contrast, the newly involved Study 2 involves several cases where each case requires sample collection from a specific region, such as the AC, TI, transverse colon (TC), descending colon (DC), sigmoid colon (SC), or rectum. Study 3 necessitates collecting samples from multiple regions. AC: ascending colon; DC: descending colon; Num.: number; REDCap: Research Electronic Data Capture; TC: transverse colon; TI: terminal ileum; SC: sigmoid colon.

The challenges identified from real-life usage were: (1) barcode robustness, (2) detailed organ-specific data storage, and (3) system performance under increased data volume that could be further improved from the published system as shown in Figure 1. To elaborate on these challenges, first, barcodes are printed at a small size of 0.92″×0.54″ using a Dymo printer with 300 dpi resolution, often becoming blurred due to accidental touch, leading to scanner recognition errors and inefficiencies in sample tracking. A more robust barcode technology is needed for better recognition by current equipment even when blurred. Second, the need to record precise sample collection locations has increased as the studies now involve collecting samples from multiple specific regions, which the previous REDCap table design does not support. Enhancing the database structure to include organ-specific fields and a flexible data entry system would improve utility in diverse studies [20]. Third, system performance degrades significantly when the REDCap record count exceeds 100, affecting data entry, query, and retrieval efficiency. Implementing database optimization techniques, such as reducing interaction times with REDCap or refining query algorithms, could maintain optimal performance as the database grows.

To address the identified issues, we propose three main design criteria for enhancing our system: (1) improving barcode robustness by updating the system with the most durable barcode technology available, (2) incorporating a dynamic organ-specific archive in the REDCap table design to support detailed anatomical data, and (3) optimizing system throughput to efficiently manage increased data volumes. Our methods include evaluating various barcode technologies under blurring conditions, implementing a dynamic organ-specific archive in REDCap, and using Docker for containerizing software clusters. In addition, we developed a local cache system to reduce interaction times with REDCap. Experimental setups involve testing barcode recognition accuracy and comparing system performance across different implementations. The results demonstrate significant improvements in barcode recognition, data granularity, and system throughput, thereby enhancing overall data management efficiency in clinical studies.

## Methods

### Overview

In this section, we detail the development and evaluation processes designed to meet the 3 proposed design criteria: improving barcode robustness, incorporating a dynamic organ-specific archive, and optimizing system throughput. Each methodology and experimental setup ensures the solutions are effective and practical for real-life clinical settings.

### Data Management Workflows Overview

**Figure 2.**
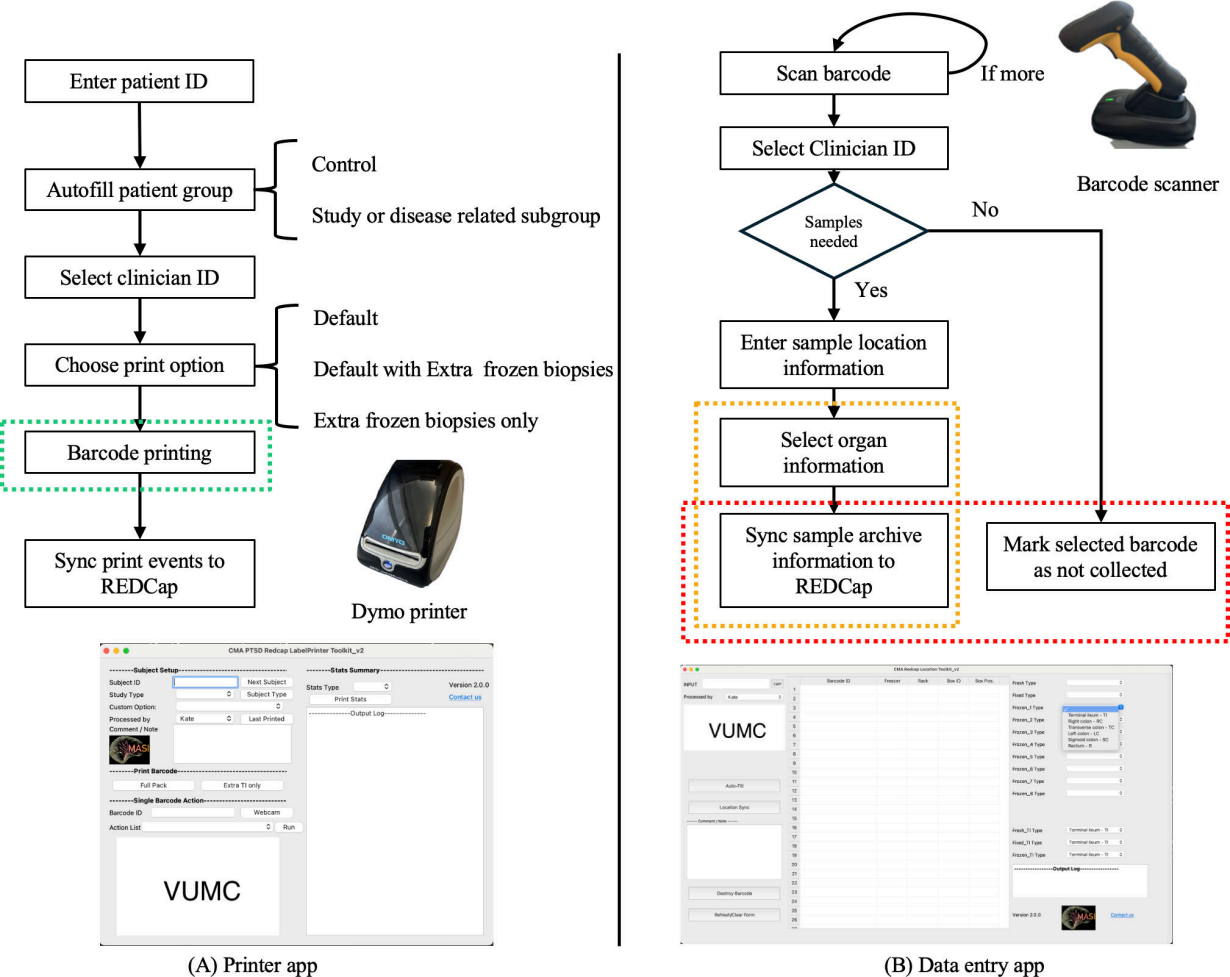
Illustration of workflows for (**A**) the Printer app and (**B**) Data entry app used in managing biopsy samples. In the Printer app, the workflow commences with entering the patient ID, followed by autofilling patient groups, selecting the clinician ID, choosing a print option, printing the barcode, and syncing print events to REDCap. The data entry app workflow involves scanning the barcode, selecting the clinician ID, determining if samples were collected, entering sample location information, selecting organ information, and either syncing sample archive information to REDCap or marking the barcode as not collected. The green, yellow, and red dotted boxes indicate the locations where systematic bottlenecks were identified in the previous implementation. These bottlenecks have been addressed and resolved in this study. REDCap: Research Electronic Data Capture; VUMC: Vanderbilt University Medical Center;

The data management workflow for 2 Python-based applications from [20], the Printer app and the Data entry app, which are integral components of our biopsy management with REDCap, is presented in Figure 2(A-B).

Briefly, the Printer app workflow begins by entering the patient ID, which autofills the patient group based on control or study or disease-related subgroups. Next, the clinician ID is selected, and a print option is chosen, ranging from default to including extra frozen biopsies. The “Barcode Printing” step, highlighted in a green box in Figure 2, was identified as a bottleneck due to limitations in printer quality and previously selected barcode design. Finally, print events are synced to REDCap, ensuring all printed barcodes are recorded in the system.

The data entry app workflow starts by scanning the barcode and selecting the clinician ID. If samples are collected, the user enters the sample location information and selects the organ information, both highlighted in yellow boxes in Figure 2. The previous system could only handle one static organ selection, which was a limitation given the diverse clinical studies we manage. If samples are not collected, the barcode is marked as not collected, and the relevant REDCap field is removed. Syncing sample archive information to REDCap, highlighted in a red box in Figure 2, is where the interaction bottleneck was found, which is critical for optimizing system throughput.

Next, we introduce design criteria based on the 3 identified bottlenecks in detail.

#### Design Criteria 1. Evaluation-Driven Barcode Robustness Improvement

**Figure 3.**
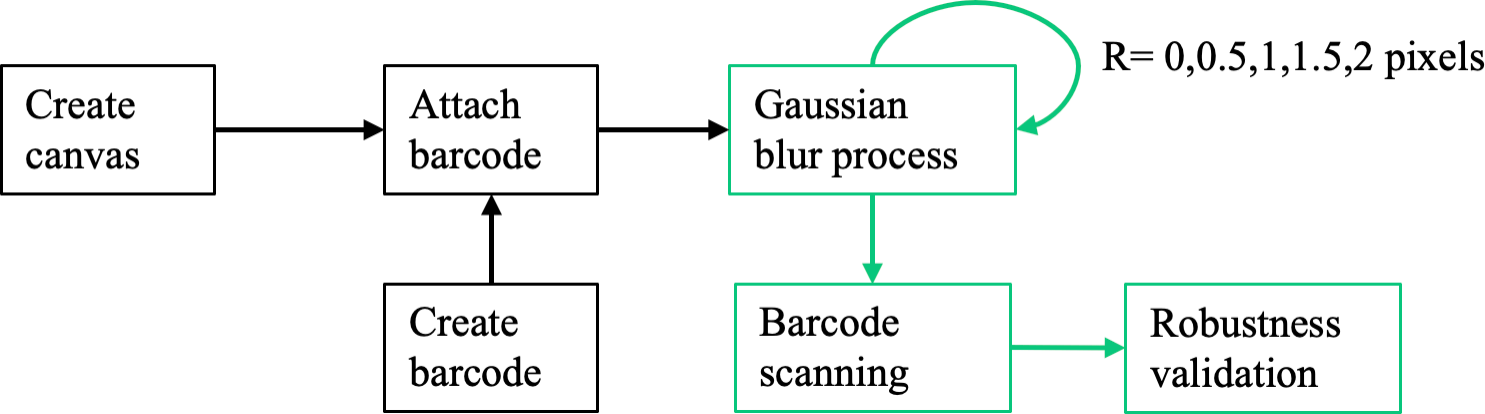
The barcode robustness evaluation process involves creating a canvas, attaching barcodes, applying a Gaussian blur with varying radii (0, 0.5, 1, 1.5, 2 pixels), scanning the barcodes, and validating their robustness.

To address the issue of barcode robustness, a series of validations (Figure 3) was conducted to assess various barcode technologies and printing methods for decoding 8-digit numerical data [20] (necessitated by the limitations of printer quality and the tiny barcode size). The experiment included evaluating the following barcode types discussed in the literature [21,22]: (1) Code 39; (2) EAN-8; (3) ITF (Interleaved 2 of 5); (4) QR Code; and (5) DataMatrix. They were chosen for their simplicity, ease of printing, and reliability in a small size.

One hundred unique 8-digit numbers were randomly generated for each of the 5 barcode types, and 5 rounds of testing were conducted. Each round applied a Gaussian blur with radii of 0, 0.5, 1, 1.5, and 2 pixels, corresponding to 0, 0.00167, 0.00333, 0.005, and 0.00667 inches, respectively. Although Gaussian blur was used as a proxy for image degradation, it does not fully capture the characteristics of printer smearing, which typically occurs in a single direction and is better modeled as motion blur. In practice, motion blur results in a point spread function resembling a rectangular function in image space and a sinc function in Fourier space. Despite this simplification, the barcodes were printed at a size of 0.94″ x0.52″, consistent with typical hospital settings. These barcodes were then scanned using the hospital’s standard scanner. The Adesso NuScan 5200TR is a 2.4GHz wireless barcode scanner (Adesso, Inc), and recognition accuracy was calculated for each barcode type and blur level. Gaussian blur was selected to systematically simulate image degradation under controlled and reproducible conditions for comparing barcode types. While this does not fully capture directional distortions such as motion blur, it enables consistent evaluation across barcodes within our experimental framework.

#### Design Criteria 2. Dynamic Organ-Specific Archive Implementation

**Figure 4.**
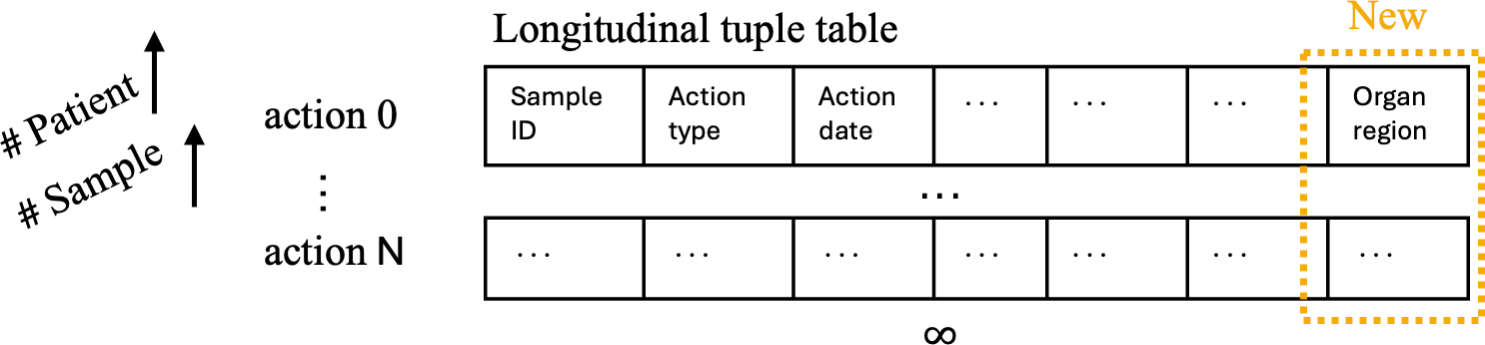
To deal with the dynamic organ-specific archival, we extend the implementation of a longitudinal tuple table with additional fields for recording the region from which each sample is collected, allowing for precise tracking of sample origins across various actions and patients.

As shown in Figure 4, the number of actions recorded in REDCap escalates rapidly with increasing patient sample collection. The design of the longitudinal tuple table was originally introduced in our previous work [20] and is briefly summarized here for completeness. In REDCap, an “arm” is a project structure that groups records either in fixed or longitudinal formats. While static arms are used for one-time data capture, longitudinal arms allow sequential event tracking over time. The longitudinal tuple table is implemented as an individual REDCap arm that flexibly records all operational actions associated with each barcode. These barcode actions include activities such as printing, scanning, storing, distributing, and destroying samples and are recorded without the need for predefined time points. This structure supports real-time, event-driven tracking and is well-suited for unpredictable and repeated operations in clinical workflows.

Previously, the system focused on biopsies collected from the ascending colon and terminal ileum [20]. In this study, we have extended the longitudinal tuple table to accommodate broader organ involvement by incorporating an organ archive, which documents the specific organ region associated with each recorded action. This enhancement allows for precise tracking of sample origins across diverse clinical protocols, particularly in studies requiring detailed anatomical localization.

The tuple table is exported in JSON format and processed using Python’s pandas library, enabling efficient filtering, querying, and consistency checks. This flexible and scalable schema aligns with REDCap’s best practices for longitudinal study designs. Further technical details can be found in our earlier conference publication [20].

In our clinical setting, the application is deployed on dedicated laboratory workstations within a controlled network. Source code is version-controlled and maintained in the MASILab GitHub group repository, which is accessible only to authorized lab members.

We use Docker to containerize three clusters of software tailored for different types of studies, including normal control, Crohn disease, colorectal cancer, ulcerative colitis, colonoscopy, and *H. pylori*-associated gastric inflammation biopsies. This containerization ensures easy deployment and consistent programming environments for each study, streamlining the process for researchers and minimizing setup complexities. To showcase the effectiveness of these updates, we provide data summaries for different projects that use the enhanced design and containerized method. We release the data dictionary design for each clinical study in the data storage section and make the relevant applications available on GitHub [23].

#### Design Criteria 3. System Throughput Optimization With Refined REDCap Interaction

**Figure 5.**
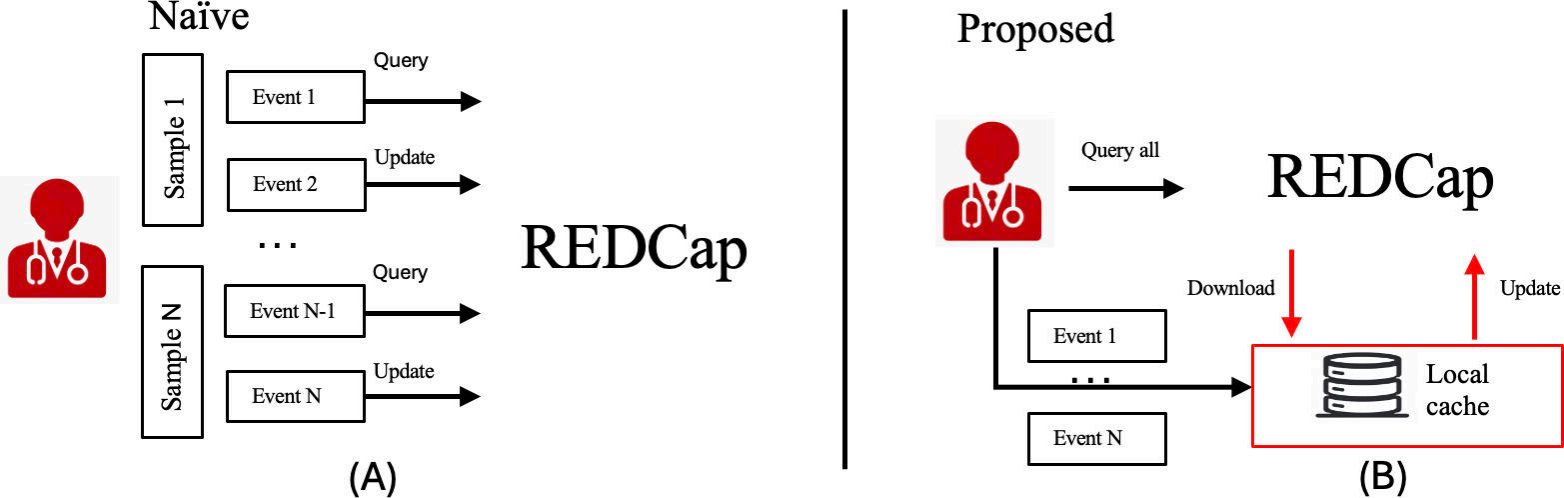
Methodological approach for system throughput optimization. (**A**) Comparison between the naïve system, (where each action involves a query and update to REDCap, and (**B**) the proposed system, which uses a local cache to reduce the number of interactions with REDCap to enhance the overall throughput. REDCap: Research Electronic Data Capture.

The throughput issue arises from continuous interactions with REDCap to download and update information. In the naïve system (Figure 5A), each sample is handled individually, performing one query and one update operation on REDCap for every sample scanned. This frequent interaction, involving verification and recording of each sample, significantly slows down the system as the number of patients and samples increases. To mitigate these interactions, we propose two approaches:

1. Bulk update action system: In this approach, the system can still perform frequent queries to REDCap but consolidates updates into a single bulk action. This method reduces the number of update operations, alleviating the bottleneck caused by frequent updates.
2. Local cache system: As illustrated in Figure 5B, a local cache method further optimizes throughput. Upon system initialization, REDCap records are downloaded into the local cache. Subsequent actions interact with the local cache instead of directly with REDCap. Once all actions are completed, the local cache is synchronized with REDCap, ensuring data integrity while minimizing interaction frequency.

To validate optimization, we designed a controlled experiment involving 500 simulated patients, each requiring 10 distinct actions to be recorded. We measured the processing time per patient sequentially, from the first to the last, and compared system performance across three different implementation designs as the number of actions increased: (1) the naïve system, (2) the bulk update action system, and (3) the local cache system.

In the naïve system, each patient’s workflow triggered 10 REDCap export application programming interface (API) calls and 10 REDCap import API calls. The bulk update action system reduced the overhead by performing 10 REDCap exports but consolidating the imports into a single REDCap import API call per patient. The local cache system further optimized the process by requiring only one REDCap export and one REDCap import API call per patient.

The purpose of this validation experiment was to provide proof-of-concept evidence that reducing the number of REDCap API calls can substantially decrease processing overhead and improve overall system throughput. This experimental setup offers a robust, systematic evaluation of how API call optimization impacts real-time data processing performance. The experiments were conducted on a workstation equipped with a 1 Gbps network connection. For each test case, we performed 5-fold evaluations and reported both the average and cumulative results with 95% CIs.

### Ethical Considerations

This project involves the use of fully de-identified data obtained from Vanderbilt University Medical Center (VUMC) under Institutional Review Board (IRB) approvals Vanderbilt IRB #191738 and #191777. While the original studies were classified as human participants research, this work is a secondary analysis focused solely on barcode system methodology and does not involve any access to protected health information (PHI) or patient identifiers by our research team. Therefore, this study does not qualify as human participants research under current regulations.

Informed consent was obtained for the primary studies at the time of patient recruitment and sample collection. The IRB approvals for these studies permit secondary analysis of deidentified data without additional consent. All data used in this study were deidentified before our analysis, ensuring that no personal health information or identifiable participant data were accessible to the study team. In addition, the barcode system uses a customized codebook that encrypts barcode information. The barcode content is only translatable within the custom-built application and cannot be independently decoded, adding an additional layer of privacy protection. No compensation was provided to participants for the studies described. No images in this manuscript or supplementary materials contain identifiable individual participants.

## Results

### Barcode Robustness Evaluation

**Figure 6.**
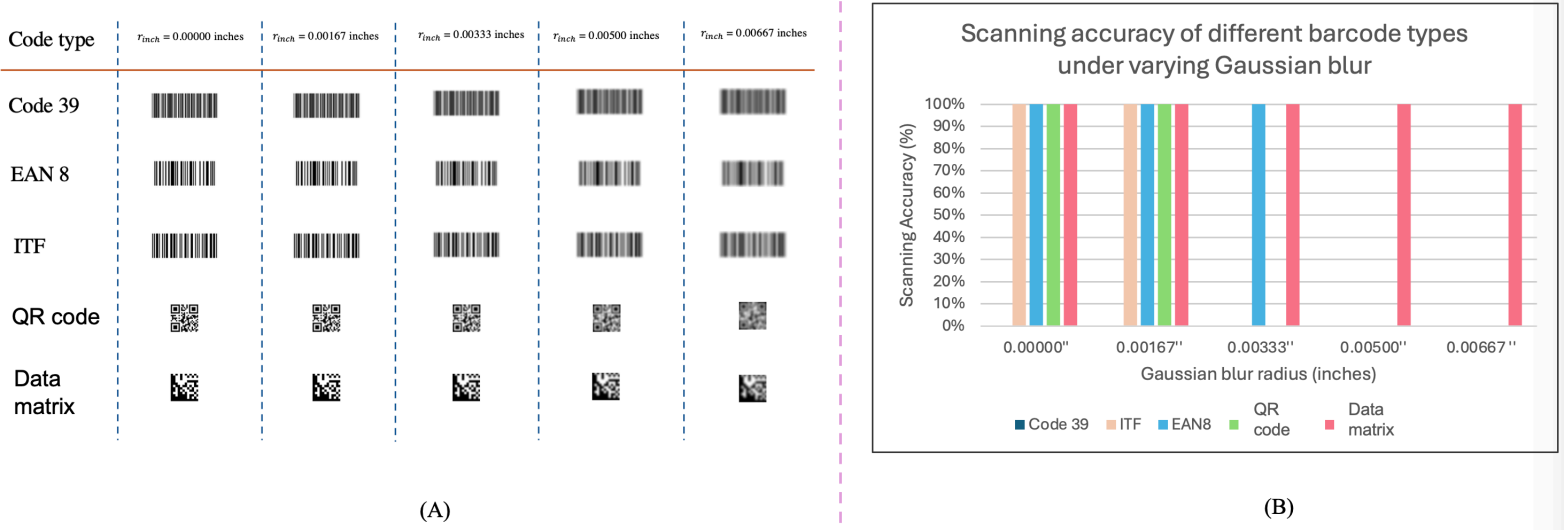
QR Code and DataMatrix show the highest resilience to blurring, maintaining near-perfect scanning accuracy across all levels of blur, while ITF and EAN-8 exhibit decreased accuracy with increasing blur levels. Code 39 failed on all validation configurations. (**A**) Examples of different barcodes subjected to varying levels of Gaussian blur. (**B**) Scanning accuracy of each barcode type under these blur conditions.

The Barcode Robustness Evaluation aims to determine which barcode technology is reliable under different blurring conditions. The experimental design, illustrated in (Figure 6A), involves subjecting each barcode type to 5 rounds of Gaussian blurring. The blur radii tested were 0, 0.5, 1, 1.5, and 2 pixels, equivalent to 0, 0.00167, 0.00333, 0.005, and 0.00667 inches, respectively. As blurring increases with each round, we tested the readability of the barcodes using a standard scanner.

The results, presented in (Figure 6B), indicate that by the fourth round, only the DataMatrix barcode remained consistently recognizable by the scanner. In contrast, the other barcode types (Code 39, EAN-8, ITF, and QR Code) failed to be accurately scanned under the same conditions. The results demonstrate that the DataMatrix barcode maintained its readability even in the final round of testing. Therefore, based on our equipment and experimental setup, we conclude that the DataMatrix barcode demonstrates the highest robustness and is the most suitable for use in our clinical environment.

### Dynamic Organ-Specific Archive Showcase

To enhance the REDCap database, we updated the table design to store organ-specific information for each action taken on a sample. This allows for precise tracking of the origin of each sample, which is essential for studies requiring detailed anatomical data.

### Demonstration of System Effectiveness Through Patient Demographics and Action Management

Following the evaluation of the 3 system designs, we present 2 tables that demonstrate the effectiveness and extensive application of our system in clinical studies. Table 1 summarizes the 4 clinical studies on recruitment procedures, target population and disease focus, recruitment locations, and recruitment time frame. For clarity, the corresponding acronyms are: CMA (Targeting Gut-Microbiome in Veterans Deployment-Related Gastrointestinal and Liver Diseases), EGD (Esophagogastroduodenoscopy project for VA_Dysregulated Polyamine), GCA (Gut Cell Atlas), and DoD (Translational Impact of Electrophile Adducts in Colorectal Cancer). As noted, the names of both CMA and DoD are not derived from the initials of their project titles but are designations aligned with clinical data collection documentation for consistency.

Together, our system covers 3 study types across 4 clinical studies:

1. CMA: Data are collected without knowing which organ is involved.
2. EGD: Data are collected from one organ, known before esophagogastroduodenoscopy. Tissues are always acquired from the gastric antrum and gastric corpus.
3. GCA: Data are collected only from the AC and TI.
4. DoD: Data are collected from one organ, known before colonoscopy.

**Table 1.** Study recruitment was conducted at multiple clinical sites depending on the study focus. The table summarizes the disease focus and recruitment time frames for each study included in this work, recruited across from VA Tennessee Valley Healthcare System, Vanderbilt University Medical Center Endoscopy, and Cooperative Human Tissue Network.

Study	Target population and disease focus
CMA^a^ (2023–present, VATVHS)^b^	Targeting gut microbiome in veterans deployment-related gastrointestinal and liver diseases: dysbiosis, posttraumatic stress disorder (PTSD), and epithelial and immune biology in inflammatory bowel disease (IBD) in veterans
EGD^c^ (2022-present**,** VATVHS)	VA_Dysregulated Polyamine Metabolism in *Helicobacter pylori* (*H. pylori*) infection and associated stomach lesions.
GCA^d^ (2019–present) VUMC^e^ Endoscopy and CHTN^f^	Combinatorial single cell strategies for a Crohn disease Gut Cell Atlas_Specimen.
DoD^g^ (2021–present) VUMC Endoscopy, CHTN, and VATVHS	Translational impact of electrophile adducts in IBD^h^ and and colorectal cancer

a CMA: a Project for Targeting Gut-Microbiome in Veterans Deployment-Related Gastrointestinal and Liver Diseases: Dysbiosis, PTSD, and Epithelial and Immune Biology in Inflammatory Bowel Disease in Veterans.

b VATVHS: VA Tennessee Valley Healthcare System.

c EGD: Esophagogastroduodenoscopy, a project for VA_Dysregulated Polyamine.

d GCA: Gut cell atlas.

e VUMC: Vanderbilt University Medical Center.

f CHTN: Cooperative Human Tissue Network.

g DoD: a project for Translational Impact of Electrophile Adducts in Colorectal Cancer.

h IBD: inflammatory bowel disease.

Table 2 demonstrates the demographic range handled by the system in terms of age, race, and gender. Summarized by Table 3, the whole studies collectively handled a total of 38,819 actions, with the GCA study alone accounting for 16,911 actions. These actions include scanning, printing, destroying, distributing, and storing samples, showcasing the system’s extensive operational capabilities. The sample types managed include fresh, fixed, and frozen tissue; serum; stool; and DNA samples for Crohn disease and ulcerative colitis. The diverse sample types and the large, significant number of actions performed—such as 5106 prints, 4092 scans, 2113 destructions, 565 distributions, and 1360 storages in the nonsurgical CD case of the GCA study—highlight the system’s flexibility and efficiency in managing different biological materials and operational tasks.

**Table 2.** A comprehensive overview of the patient demographics involved in various clinical studies using our system.

Study name and surgery		Study case	Patient, n/N (%)	Age, mean (SD)	Gender, n/N (%)			Race, n/N (%)				
					Male	Female	N/A^a^	Asian	Black/African American	White/Caucasian	Hispanic /Latino	N/A^a^
CMA^b^												
No			34/34 (100)	48.6 (2.0)	27/34 (80)	5/34 (15)	2/34 (6)	0/34 (0)	3/34 (9)	29/34 (85)	0/34 (0)	2/34 (6)
EGD^c^												
No			52/52 (100)	56.2 (2.0)	45/52 (87)	5/52 (10)	2/52 (3)	1/52 (2)	17/52 (33)	32/52 (62)	0/52 (0)	2/52 (3)
GCA^d^												
No		CD^e^	125/179 (70)	36.1 (1.3)	47/179 (26)	78/179 (44)	0/179 (0)	4/179 (2)	13/179 (7)	106/179 (60)	2/179 (1)	0/179 (0)
No		Control	41/179 (23)	54.9 (1.0)	16/179 (8)	25/179 (14)	3/179 (1)	1/179 (1)	3/179 (1)	36/179 (20)	1/179 (1)	0/179 (0)
Yes		CD	10/179 (6)	N/A	0/179 (0)	0/179 (0)	10/179 (6)	0/179 (0)	0/179 (0)	0/179 (0)	0/179 (0)	10/179 (6)
Yes		Control	3/179 (1)	N/A	0/179 (0)	0/179 (0)	3/179 (1)	0/179 (0)	0/179 (0)	0/179 (0)	0/179 (0)	3/179 (1)
DOD^f^												
No		CD	38/144 (26)	38.9 (2.5)	15/144 (10)	23/144 (16)	0/144 (0)	0/144 (0)	2/144 (1)	34/144 (24)	0/144 (0)	2/144 (1)
No		Control	19/144 (13)	52.9 (2.9)	13/144 (9)	6/144 (4)	0/144 (0)	1/144 (1)	0/144 (0)	15/144 (10)	0/144 (0)	3/144 (2)
No		UC^g^	50/144 (35)	41.0 (2.0)	23/144 (16)	27/144 (18)	0/144 (0)	0/144 (0)	2/144 (1)	43/144 (30)	1/144 (1)	4/144 (3)
Yes		CD	19/144 (13)	N/A	0/144 (0)	0/144 (0)	0/144 (0)	19/144 (13)	0/144 (0)	0/144 (0)	0/144 (0)	19/144 (13)
Yes		Control	1/144 (1)	N/A	0/144 (0)	0/144 (0)	0/144 (0)	1/144 (1)	0/144 (0)	0/144 (0)	0/144 (0)	1/144 (1)
Yes		UC	5/144 (3)	N/A	0/144 (0)	0/144 (0)	0/144 (0)	5/144 (3)	0/144 (0)	0/144 (0)	0/144 (0)	5/144 (3)
Yes		Cancer	12/144 (8)	N/A	0/144 (0)	0/144 (0)	0/144 (0)	12/144 (8)	0/144 (0)	0/144 (0)	0/144 (0)	12/144 (8)

a N/A: not available.

b CMA: project for Targeting Gut-Microbiome in Veterans Deployment-Related Gastrointestinal and Liver Diseases: Dysbiosis, PTSD, and Epithelial and Immune Biology in Inflammatory Bowel Disease in Veterans.

c EGD: Esophagogastroduodenoscopy, a project for VA_Dysregulated Polyamine.

d GCA: Gut Cell Atlas.

e CD: Crohn disease.

f DOD: Project for translational impact of electrophile adducts in colorectal cancer.

g UC: ulcerative colitis.

**Table 3.** Various actions and sample types managed by our system across different studies.

Study name and surgery	Study case	Action	Sample type						Action type				
Fresh	Fixed	Frozen	Serum	Stool	DNA	Printed	Scanned	Destroyed	Distributed	Stored
CMA,^a^ n/N (%)													
No		3819/3819 (100)	278/3819 (7)	266/3819 (7)	1222/3819 (32)	1560/3819 (41)	493/3819 (13)	0/3819 (0)	1456/3819 (38)	1087/3819 (28)	271/3819 (7)	94/3819 (2)	911/3819 (24)
EGD,^b^ n/N (%)													
No		1372/1372 (100)	343/1372 (25)	313/1372 (23)	716/1372 (52)	0/1372 (0)	0/1372 (0)	0/1372 (0)	481/1372 (35)	439/1372 (32)	37/1372 (3)	206/1372 (1)	209/1372 (1)
GCA,^c^ n/N (%)													
No	CD^d^	13236/16908 (78)	1733/16908 (10)	1877/16908 (11)	2385/16908 (14)	5176/16908 (31)	1617/16908 (10)	448/16908 (3)	5106/16908 (30)	4092/16908 (24)	2113/16908 (12)	565/16908 (3)	1360/16908 (8)
No	Control	3288/16908 (19)	218/16908 (1)	306/16908 (2)	449/16908 (3)	1637/16908 (10)	548/16908 (3)	130/16908 (1)	1085/16908 (6)	1145/16908 (7)	290/16908 (2)	204/16908 (1)	564/16908 (3)
Yes	CD	203/16908 (1)	62/16908 (0)	65/16908 (0)	76/16908 (0)	0/16908 (0)	0/16908 (0)	0/16908 (0)	63/16908 (0)	72/16908 (0)	5/16908 (0)	40/16908 (0)	23/16908 (0)
Yes	Control	181/16908 (1)	58/16908 (0)	57/16908 (0)	66/16908 (0)	0/16908 (0)	0/16908 (0)	0/16908 (0)	48/16908 (0)	84/16908 (0)	0/16908 (0)	32/16908 (0)	17/16908 (0)
DOD,^e^ n													
No	CD	2374/9076 (26)	135/9076 (1)	133/9076 (1)	419/9076 (5)	1556/9076 (17)	0/9076 (0)	131/9076 (1)	868/9076 (10)	788/9076 (9)	129/9076 (1)	114/9076 (1)	475/9076 (5)
No	Control	1366/9076 (15)	78/9076 (1)	75/9076 (1)	241/9076 (3)	897/9076 (10)	0/9076 (0)	75/9076 (1)	582/9076 (6)	409/9076 (5)	62/9076 (1)	60/9076 (1)	253/9076 (3)
No	UC^f^	3371/9076 (37)	186/9076 (2)	197/9076 (2)	683/9076 (8)	2118/9076 (23)	0/9076 (0)	187/9076 (2)	1161/9076 (13)	1142/9076 (13)	227/9076 (3)	157/9076 (2)	684/9076 (8)
Yes	CD	819/9076 (9)	122/9076 (1)	122/9076 (1)	506/9076 (6)	11/9076 (0)	0/9076 (0)	58/9076 (1)	292/9076 (3)	271/9076 (3)	107/9076 (1)	56/9076 (1)	93/9076 (1)
Yes	Control	186/9076 (2)	30/9076 (0)	30/9076 (0)	111/9076 (1)	0/9076 (0)	0/9076 (0)	15/9076 (0)	102/9076 (1)	52/9076 (1)	1/9076 (0)	10/9076 (0)	21/9076 (0)
Yes	UC	425/9076 (5)	64/9076 (1)	65/9076 (1)	257/9076 (3)	11/9076 (0)	0/9076 (0)	28/9076 (0)	169/9076 (2)	106/9076 (1)	32/9076 (0)	37/9076 (0)	81/9076 (1)
Yes	Cancer	535/9076 (6)	96/9076 (1)	98/9076 (1)	295/9076 (3)	0/9076 (0)	0/9076 (0)	46/9076 (1)	183/9076 (2)	169/9076 (2)	74/9076 (1)	56/9076 (1)	52/9076 (1)

a CMA: Project for Targeting Gut-Microbiome in Veterans Deployment Related Gastrointestinal and Liver Diseases: Dysbiosis, PTSD, and Epithelial and Immune Biology in Inflammatory Bowel Disease in Veterans.

b EGD: Esophagogastroduodenoscopy, a project for VA_Dysregulated Polyamine.

c GCA: Gut Cell Atlas.

d CD: Crohn disease.

e DOD: Project for translational impact of electrophile adducts in colorectal cancer.

f UC: ulcerative colitis.

### System Throughput Optimization With Refined REDCap Interaction

**Figure 7.**
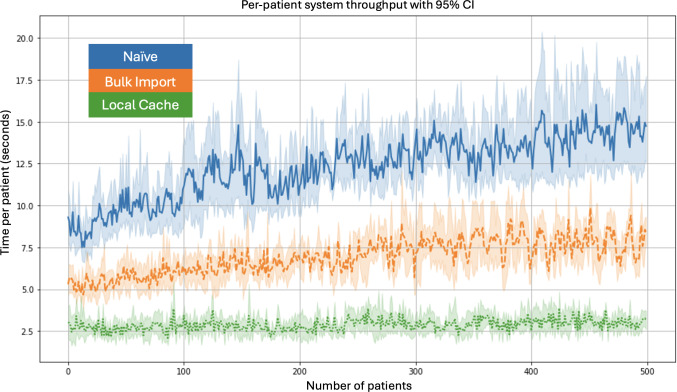
Per-patient system throughput across 3 implementations with 95% CIs. The naïve system shows increasing processing time per patient as the number of patients grows. The bulk import system reduces this overhead, while the local cache system maintains the lowest and most stable processing time per patient across all patient counts.

The system throughput evaluation demonstrated consistent performance differences across the 3 implementations. Pairwise 2-tailed *t* tests were conducted, demonstrating that the performance differences between test cases were statistically significant (*P*<.001). As shown in Figure 7**,** the naïve system’s per-patient processing time steadily increased with the number of patients, largely due to the accumulation of overhead from multiple REDCap API calls (10 export and 10 import calls per patient). The bulk import system effectively reduced this overhead by consolidating import operations, resulting in a lower and more stable per-patient processing time. The local cache system exhibited the most efficient performance, maintaining minimal and stable processing times across all patient counts by further reducing the API call frequency to one export and one import per patient.

**Figure 8.**
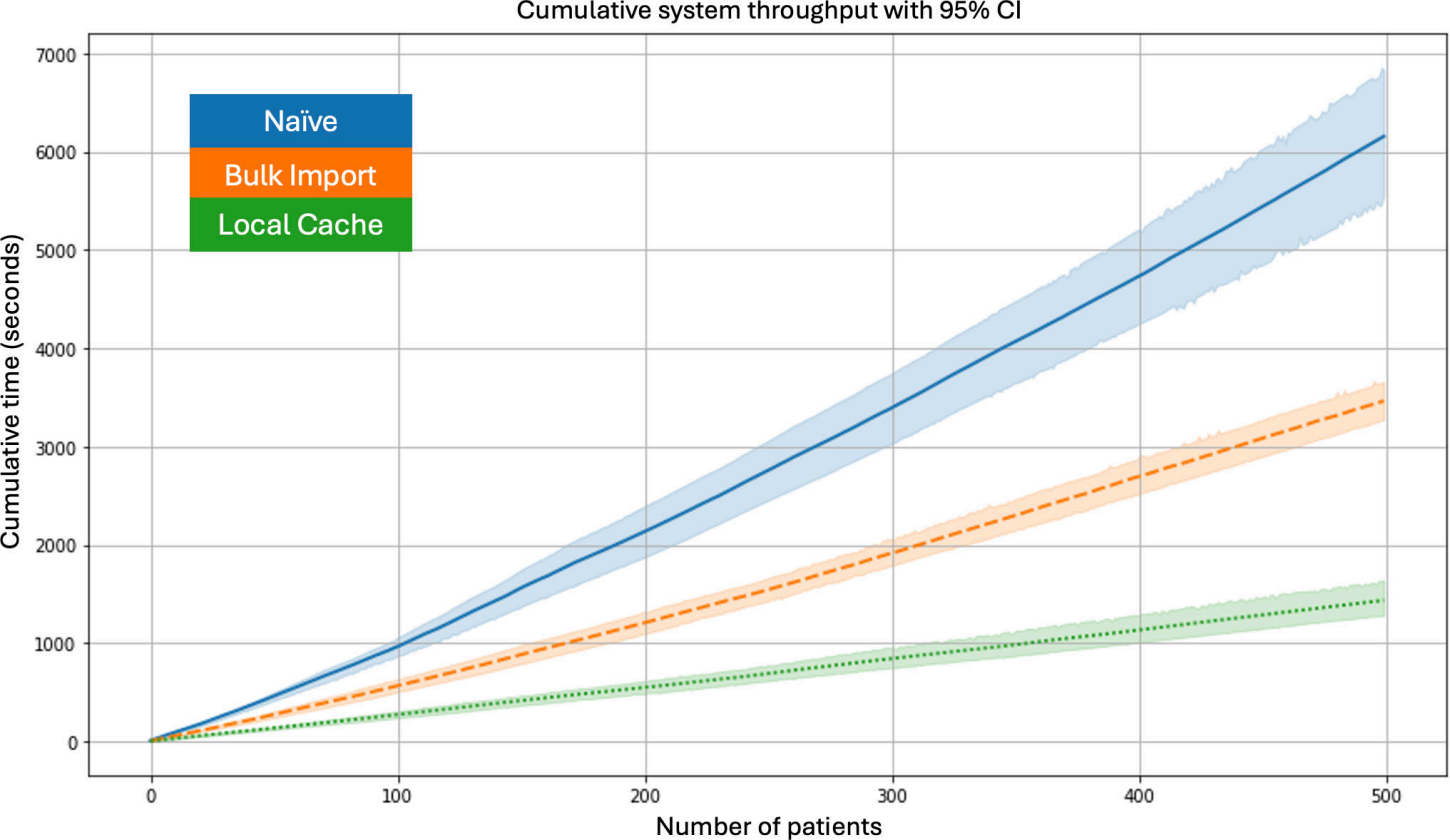
Cumulative system throughput across 3 implementations with 95% CIs. The naïve system exhibits the steepest cumulative processing time curve. The bulk import system reduces cumulative load, while the local cache system achieves the lowest cumulative processing time, demonstrating superior scalability.

The cumulative processing time, presented in Figure 8, further underscores the efficiency gains of the optimized systems. The naïve system displayed the steepest cumulative growth, reflecting its scalability limitations. In contrast, the bulk import system achieved moderate improvement, and the local cache system substantially minimized cumulative processing time, demonstrating superior scalability and throughput. These results validate the hypothesis that reducing the number of REDCap API calls is critical for improving system efficiency and highlight the robustness of the local cache approach for large-scale clinical workflows.

## Discussion

### Overview

In this study, we aimed to enhance clinical data management by improving barcode robustness, increasing data granularity, and optimizing system throughput to better support real-world clinical workflows. Our results demonstrated that the DataMatrix barcode provided superior recognition accuracy under blurred conditions, addressing barcode robustness issues identified in previous work. By incorporating a dynamic organ-specific archive within REDCap, we enabled precise tracking of sample origins, supporting studies with complex and evolving anatomical requirements. In addition, system throughput was significantly improved through the introduction of a local cache mechanism, reducing REDCap API interaction overhead and increasing processing efficiency. Together, these enhancements address key bottlenecks in barcode informatics systems and validate the effectiveness of the proposed design criteria across diverse gastrointestinal clinical studies. In the following subsections, we provide a detailed discussion of these findings, including comparisons to previous work and an evaluation of system limitations.

### Principal Results

The enhancements proposed and tested in this study significantly improve the efficiency and robustness of the cross-platform informatics system initially developed by Bao et al [20]. By addressing key issues such as barcode robustness, detailed data archiving, and system throughput, we have demonstrated tangible benefits in managing clinical data. The DataMatrix barcode was identified as the most reliable under various blurring conditions, ensuring accurate sample tracking even in suboptimal environments.

Although both QR Code and DataMatrix barcodes demonstrated high resilience under blurring, our final selection of the DataMatrix barcode was driven by the practical constraints of our clinical workflow. Specifically, the DYMO LabelWriter 450 printer (~US$200) used in our system could not consistently produce QR codes with sufficient resolution on the Diversified Biotech DTCR-6000 Direct Thermal Cryo-Tags (Sigma-Aldrich; 5″×1.5″, US$45.50 per roll of 500), which are required to fit 1.5 mL cryovials. DataMatrix barcodes are more space-efficient and less sensitive to printer resolution, enabling reliable scanning at this small label size. The Adesso NuScan 5200TR scanner (~US$200) used in our system was capable of scanning both barcode types robustly, confirming that scanner limitations were not the primary issue.

The implementation of a dynamic organ-specific archive in REDCap allows for precise tracking of sample origins, enhancing data granularity and usability. Organ selection within our system is guided by the clinical protocols of each study, which define the sampling regions in advance. Given that each project may target different anatomical sites, the system allows flexibility in specifying the organ for each action. In cases where ambiguous or overlapping anatomical regions arise, both the printer app, data entry app, and the REDCap schema include dedicated comment fields that enable clinicians to record relevant details or uncertainties. It is important to note that the barcode system serves as a tracking mechanism and does not resolve anatomical ambiguities; instead, such contextual details are manually documented to ensure clinical accuracy.

In clinical practice, evolving study protocols and mid-study changes in data collection requirements are common. Our system is designed to accommodate some of these changes, such as adding new label types or supporting additional sample collection strategies (eg, switching to frozen biopsies when fresh samples are unavailable) without disrupting the existing workflow. More substantial protocol changes, such as significant modifications in label design or data entry workflows, may require the deployment of new application versions. Our framework supports this by allowing distinct study cases with customized printing and data entry workflows. One key lesson learned is the value of improving label printing flexibility to enable on-the-fly adaptations in future versions of the system.

REDCap’s built-in caching mechanisms, such as project dashboard caching, are designed to improve web interface performance by temporarily storing data in the browser when records remain unchanged. These optimizations do not apply to API-based workflows or external applications that require real-time, high-frequency interactions, such as barcode scanning and sample tracking. Our system introduces a local cache layer that supports customized, app-integrated workflows, significantly reducing the need for repetitive API calls and improving throughput during barcode-driven operations, ensuring smooth operation even with large patient datasets. These improvements collectively enhance the system’s ability to support complex and large-scale clinical studies, thereby contributing to more reliable and efficient data management practices.

### Comparison With Previous Work

Our work builds on the foundation established by previous work Bao et al [20], introducing significant improvements in barcode technology, data granularity, and system throughput.

The system incorporates practical error-handling mechanisms for both barcode scanning and REDCap database accessibility. In early versions [20], we observed occasional scanning failures with the EAN-8 barcode format, which led us to adopt the more robust DataMatrix format in the current system. No barcode scanning failures have been observed since this transition. In addition, the application supports manual barcode entry to handle any potential scanner-related issues.

While the original system provided a complete framework for clinical data management, our enhancements address specific bottlenecks identified in real-world usage. Previous studies have highlighted the importance of reliable barcode tracking and data entry consistency in clinical settings, but our study uniquely combines these aspects with a comprehensive approach to system optimization, demonstrating substantial performance gains. The scalability evaluation using 500 patients and 10 actions per patient was designed as a proof-of-concept to assess system performance under moderate workload conditions. The primary performance constraint arises from the frequency and volume of REDCap API interactions, which are tightly coupled with system throughput. While the current local cache mechanism significantly reduces API traffic, scaling the system to support significantly larger datasets would require further architectural optimization, including improved cache synchronization, enhanced concurrency handling, and more systematic pressure testing to assess system robustness at scale. Exploring these enhancements will be essential to support high-volume clinical studies in future deployments.

### Limitations

Our barcode robustness assessment did not directly incorporate motion blur or real-world scanner variability. Future studies should address these factors to further validate barcode performance in practical clinical environments. In addition, the evaluation of barcode robustness was conducted under controlled conditions, which may not fully capture the broader variability of real-world clinical environments. The system’s performance and usability could also benefit from further enhancements to the user interface to better accommodate a wider range of clinical study applications.

In cases where the REDCap database becomes temporarily inaccessible, the system either prevents application launch (as a REDCap record export is required during initialization) or triggers a warning message if a connection failure occurs during active operation. While these mechanisms provide basic error notifications, further improvements in offline operation support and automatic recovery strategies will be considered in future work.

Despite these, the local cache mechanism, while effective, is currently designed for use by a single operator at a time. This may limit its applicability in larger research settings where multiple operators need to work simultaneously. Specifically, the system supports a text-based flag to prevent simultaneous access by multiple users on the same workstation; this feature is not enabled in the production environment. In clinical practice, strict single-user operation per workstation is enforced to maintain data integrity and simplify workflow management. Multi-user concurrency across different workstations is not supported in the current system. Supporting such functionality would require robust cross-machine locking, cache synchronization, and efficient REDCap API management, which remains an important area for future development.

Furthermore, data integrity between the local cache and REDCap is safeguarded by ensuring that the local cache is updated only after a successful sync process is completed. If the system fails to retrieve updated REDCap records, a warning message is triggered to alert the user of the issue. However, this safeguard has not been systematically validated under high-volume or failure-prone conditions. Future work will focus on more rigorous testing of the sync process, including controlled failure scenarios and large-scale data loads, to ensure cache consistency and system resilience.

Finally, Docker containers in this work are provided to support users who wish to test the system or replicate the design criteria but are not the primary security mechanism. Future work will explore strategies for securely deploying this system across multiple research teams and sites.

### Conclusions

The proposed enhancements significantly improve barcode robustness, data granularity, and system throughput in our informatics system, enabling more efficient and reliable clinical data management. These optimizations not only support diverse clinical research workflows but also demonstrate a scalable framework that can be adapted for complex, multiorgan studies across different institutions. Beyond the immediate performance gains, this work highlights the importance of system design strategies that minimize external API dependencies to achieve throughput improvements in high-volume clinical settings. Looking forward, future enhancements will focus on developing synchronization capabilities to support simultaneous multioperator use and creating a more versatile user interface to accommodate a broader range of study designs. These advances will further expand the system’s applicability and promote its integration into larger, multisite research environments.

## Abbreviation

AC: ascending colon
API: application programming interface
CD: Crohn disease
CHTN: Cooperative Human Tissue Network
CMA: Project for Targeting Gut-Microbiome in Veterans Deployment Related Gastrointestinal and Liver Diseases: Dysbiosis, PTSD, and Epithelial and Immune Biology in Inflammatory Bowel Disease in Veterans.
DC: descending colon
DoD: Project for Translational Impact of Electrophile Adducts in Colorectal Cancer
EGD: esophagogastroduodenoscopy
GCA: Gut Cell Atlas
*H. pylori*: *Helicobacter pylori*
IBD: inflammatory bowel disease
ITF: Interleaved 2 of 5
PTSD: posttraumatic stress disorder
REDCap: Research Electronic Data Capture
SC: sigmoid colon
TC: transverse colon
TI: terminal ileum
UC: ulcerative colitis
VATVHS: VA Tennessee Valley Healthcare System
VUMC: Vanderbilt University Medical Center
